# Intracranial Cryptococcoma Mimicking Stroke or Brain Tumor: A Diagnostic Challenge in an Immunocompetent Patient

**DOI:** 10.7759/cureus.82630

**Published:** 2025-04-20

**Authors:** Ryotaro Otsuka, Taro Komuro, Haruki Yamashita, Yuto Mitsuno, Manabu Kurosawa, Satoshi Horiguchi

**Affiliations:** 1 Department of Neurosurgery, Nagahama City Hospital, Nagahama, JPN; 2 Department of Neurosurgery, Kyoto University Graduate School of Medicine, Kyoto, JPN; 3 Department of Pathology, Nagahama City Hospital, Nagahama, JPN

**Keywords:** central cryptococcal infection, cryptococcoma, immunocompetent patients, stroke mimics, tumor mimics

## Abstract

Central nervous system (CNS) cryptococcal infections can manifest as meningitis in immunocompromised patients. Although CNS cryptococcal infections are rare in immunocompetent individuals, when they do occur, they often present as cryptococcomas rather than meningitis. We report a case of an immunocompetent patient who was initially suspected of cerebral infarction but was finally diagnosed with a central cryptococcal infection, highlighting the challenges of diagnosing it in these patients and the importance of early intervention.

A 58-year-old immunocompetent man with a prior transient ischemic attack and stroke presented with transient dysarthria and right upper extremity paralysis, initially raising suspicion of cerebral infarction. However, atypical findings on diffusion-weighted imaging (DWI) and pronounced cerebral edema prompted further evaluation. Contrast-enhanced magnetic resonance imaging (MRI) showed a ring-enhancing lesion, raising suspicion of a brain tumor. Biopsy confirmed an unexpected diagnosis of cryptococcosis. Further history-taking revealed that the patient worked in a factory handling agricultural equipment, which was frequently visited by pigeons, a known risk factor for cryptococcal exposure. The patient was treated with antifungal therapy and discharged.

Immunocompetent patients with central cryptococcal infections that mimic other conditions, such as stroke or brain tumors, may present diagnostic challenges. Detailed history taking, symptom evaluation, and accurate imaging interpretation are crucial for timely diagnosis and intervention. Early antifungal therapy is crucial and highlights the importance of considering cryptococcal infections even if atypical symptoms or misleading MRI findings are present.

## Introduction

Cryptococcosis is a systemic fungal infection caused by *Cryptococcus neoformans *or* Cryptococcus gattii*. *Cryptococcus* can cause a wide range of diseases, from asymptomatic pulmonary lesions to disseminated infections affecting multiple organs, particularly the central nervous system (CNS). CNS cryptococcal infections often manifest as meningitis in immunocompromised patients, including those with human immunodeficiency virus (HIV); however, this condition is rare in immunocompetent individuals [1-3]. In immunocompetent patients, central cryptococcal infections result in cryptococcoma formation, which is a granulomatous reaction, rather than meningitis [4]. These infections may cause focal symptoms or epileptic seizures and may be misdiagnosed as brain tumors on contrast-enhanced magnetic resonance imaging (MRI) [5-7]. In this report, we describe a case of cryptococcoma in an immunocompetent patient who was initially suspected of having cerebral infarction based on symptoms and diffusion-weighted imaging (DWI) findings, and contrast-enhanced MRI raised suspicion of a brain tumor. Ultimately, a craniotomy with biopsy was performed, and histopathological examination (HPE) confirmed the diagnosis of a central nervous system cryptococcal infection with cryptococcoma.

This is an important case as it suggests that detailed history taking, symptom evaluation, accurate MRI assessment, and an appropriate pathological diagnosis by biopsy can accurately diagnose a central cryptococcal infection with cryptococcoma in immunocompetent patients, resulting in timely antifungal treatment that may improve prognosis. However, cryptococcal infections, particularly cryptococcal meningitis, are associated with high mortality due to increased intracranial pressure (ICP). Additionally, immune reconstitution inflammatory syndrome (IRIS) can occur during treatment, requiring careful monitoring.

This case is not exclusive to large-scale institutions such as academic hospitals and is valuable for all physicians as this condition should not be missed and can also be encountered in general hospitals.

## Case presentation

**Figure 1:**
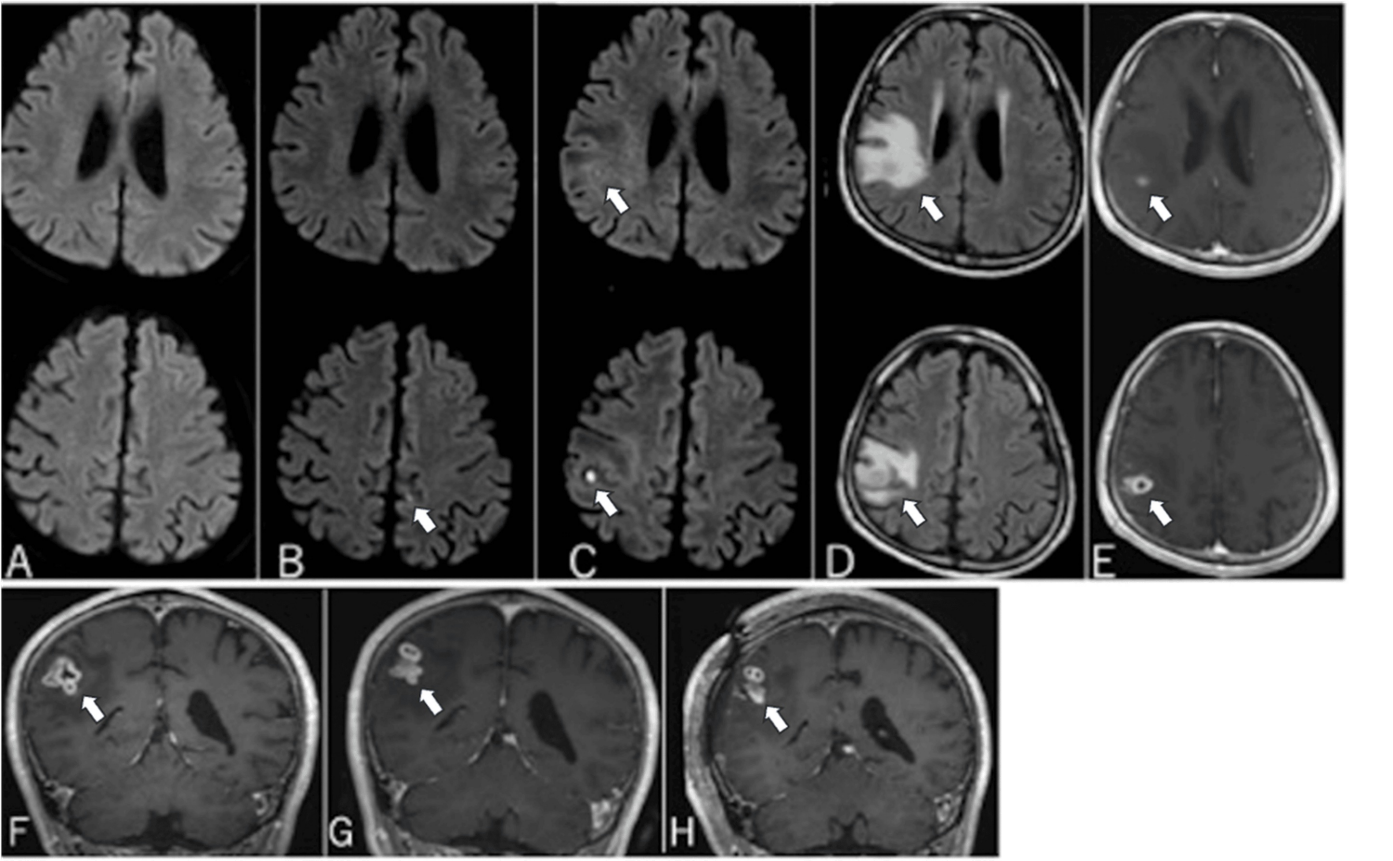
Cranial MRI (A-C: DWI, D: FLAIR, E-I: contrast-enhanced MRI). (A) Initial presentation with a transient ischemic attack (TIA); no abnormal findings were observed. (B) At 6 months after the initial visit, revealing cerebral infarction (arrowhead) in the left parietal lobe. (C) At 11 months after the initial visit, showing a hyperintense signal on DWI in the right parietal lobe (arrowhead), with findings atypical for cerebral infarction. (D) A FLAIR image was obtained showing extensive brain edema surrounding the lesion (arrowhead). (E) A contrast-enhanced MRI was obtained after admission, revealing a ring-enhancing lesion in the right parietal lobe (arrowhead). (F) Upon admission, with a contrast-enhancing lesion (arrowhead). (G) Immediately before biopsy, the contrast-enhancing lesion was slightly enlarged (arrowhead).(H) Post-biopsy MRI showed that a small amount of the lesion was removed by biopsy (arrowhead) and that there were no surgical complications. MRI: Magnetic resonance Iimaging, DWI: Diffusion-weighted imaging, FLAIR: Fluid-attenuated inversion recovery, TIA: Transient ischemic attack

A 58-year-old immunocompetent man (BMI: 20.3 kg/m²) without a history of opportunistic infections or disease and who was not taking any regular medication presented to our hospital with dysarthria, numbness, and weakness of his right upper extremity. Notably, these symptoms were transient. A cranial MRI revealed no evidence of a cerebral infarction or hemorrhage (Figure 1A). Thus, the patient was diagnosed with a transient ischemic attack (TIA) and was admitted for treatment. Following discharge, the patient returned to the hospital six months later due to dysarthria and weakness of the right upper and lower limbs. A cranial MRI revealed a small cerebral infarction in the left parietal lobe (Figure 1B). Following hospitalization and treatment, the patient’s symptoms resolved.

The patient again presented to the hospital 11 months after the initial episode, complaining of weakness of the left upper limb and dysarthria. The patient did not have a fever. A cranial MRI showed a hyperintense lesion on DWI in the right parietal lobe surrounded by edematous changes (Figure 1C, 1D). As the DWI results were atypical for cerebral infarction, a contrast-enhanced MRI was performed, revealing a ring-enhancing lesion in the right parietal lobe (postcentral gyrus). Subsequently, the differential diagnoses included metastatic brain tumor, glioblastoma, and brain abscess (Figures 1E, 1F). Contrast-enhanced computed tomography (CT) imaging of the chest and abdomen showed no evidence of tumor or abscess lesions. Blood and cerebrospinal fluid culture results were negative. Results of tests for infectious diseases, including HIV screening, were negative, and levels of tumor markers were normal. Immunological testing revealed normal serum complement levels (C3: 114 mg/dL, C4: 26.3 mg/dL, CH50: within normal range) and total IgG levels (1252 mg/dL), suggesting no significant complement deficiency or hypogammaglobulinemia. However, additional assessments, such as lymphocyte subset analysis and immunoglobulin subclass measurements, were not performed. Other tests, including beta-D-glucan, and soluble interleukin-2 receptor, were also negative.

Two weeks after admission, a craniotomy was performed to further evaluate the lesion, revealing that the arachnoid membrane and brain surface beneath appeared opaque. The lesion, which was distinguishable from the surrounding normal brain tissue, appeared white. Intra-operative pathology consultation indicated the absence of malignant lesions, suggesting an inflammatory condition or granulomatous lesion. As the lesion’s location was in the postcentral gyrus, excision was considered potentially harmful. Consequently, the surgery was concluded with only a biopsy being performed (Figures 1G, 1H).

**Figure 2:**
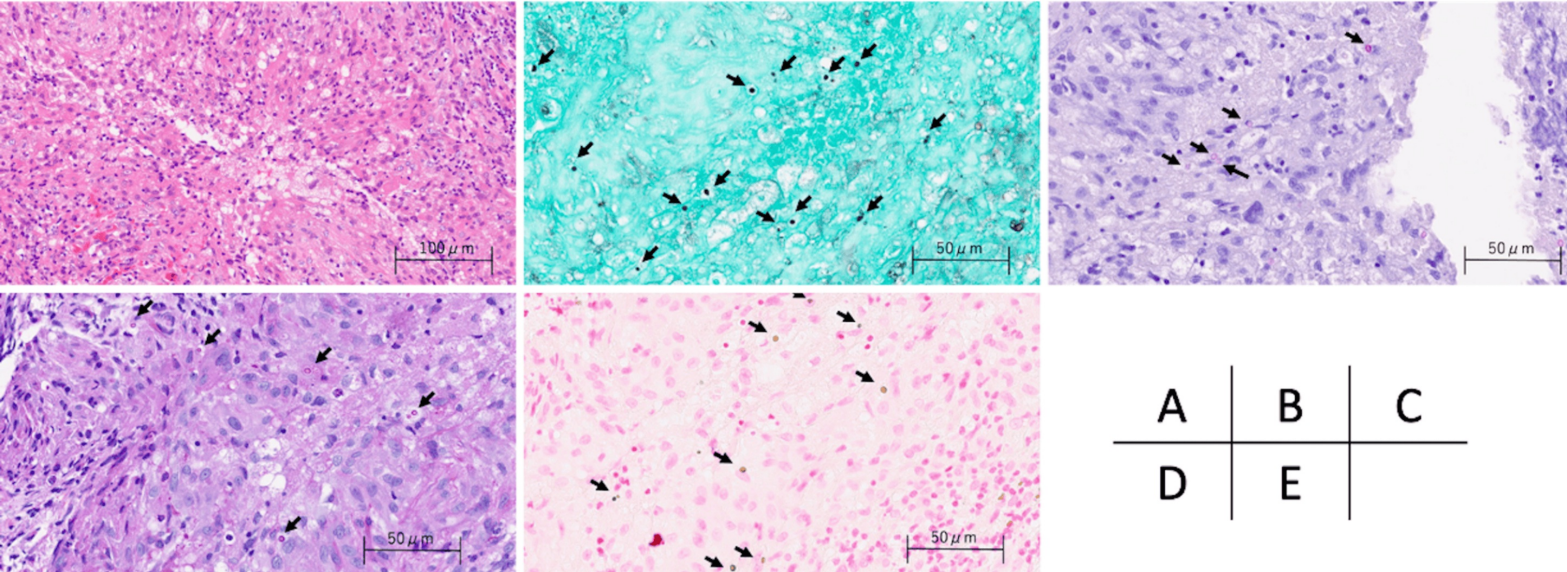
Histopathological analysis of the lesion (arrow shows Cryptococci) (HE is 200x, all others are 400x). Mucicarmine staining confirmed the presence of capsules, while periodic acid-Schiff, Grocott’s, and Fontana-Masson staining were positive for yeast-like fungi. (A) hematoxylin and eosin stain.(B) Grocott stain. (C) mucicarmine staining. (D) periodic acid-Schiff stain. (E) Masson–Fontana staining.

According to additional pathologic studies, periodic acid-Schiff (PAS), Grocott’s, and Fontana-Masson staining were positive for yeast-like fungi. Mucicarmine staining confirmed the presence of capsules (Figure 2).

The cerebrospinal fluid obtained during surgery was negative for fungal infections. CSF analysis showed total protein: 350 mg/dL, glucose: 33 mg/dL, and WBC count: 183 cells/μL. These findings were consistent with cryptococcal meningitis, as they demonstrated markedly elevated protein, low glucose, and pleocytosis, which are characteristic of fungal CNS infections. Although bacterial brain abscesses may present with similar findings, the moderate elevation of WBC count and the presence of a granulomatous lesion on histopathology supported a presumed diagnosis of cryptococcoma as the primary etiology. Accordingly, a presumed diagnosis of cryptococcoma, namely a granulomatous lesion caused by *Cryptococcus* infection, was established. Interestingly, the patient reported a history of numerous pigeons frequenting the factory premises where he worked. Treatment was initiated 26 days after MRI identification and following a presumed pathological diagnosis with amphotericin B (L-AMB) (300 mg per day) and 5-fluorocytosine (5-FC) (4,000 mg per day). One week later, a contrast-enhanced MRI showed a reduction in the lesion size.

**Figure 3:**
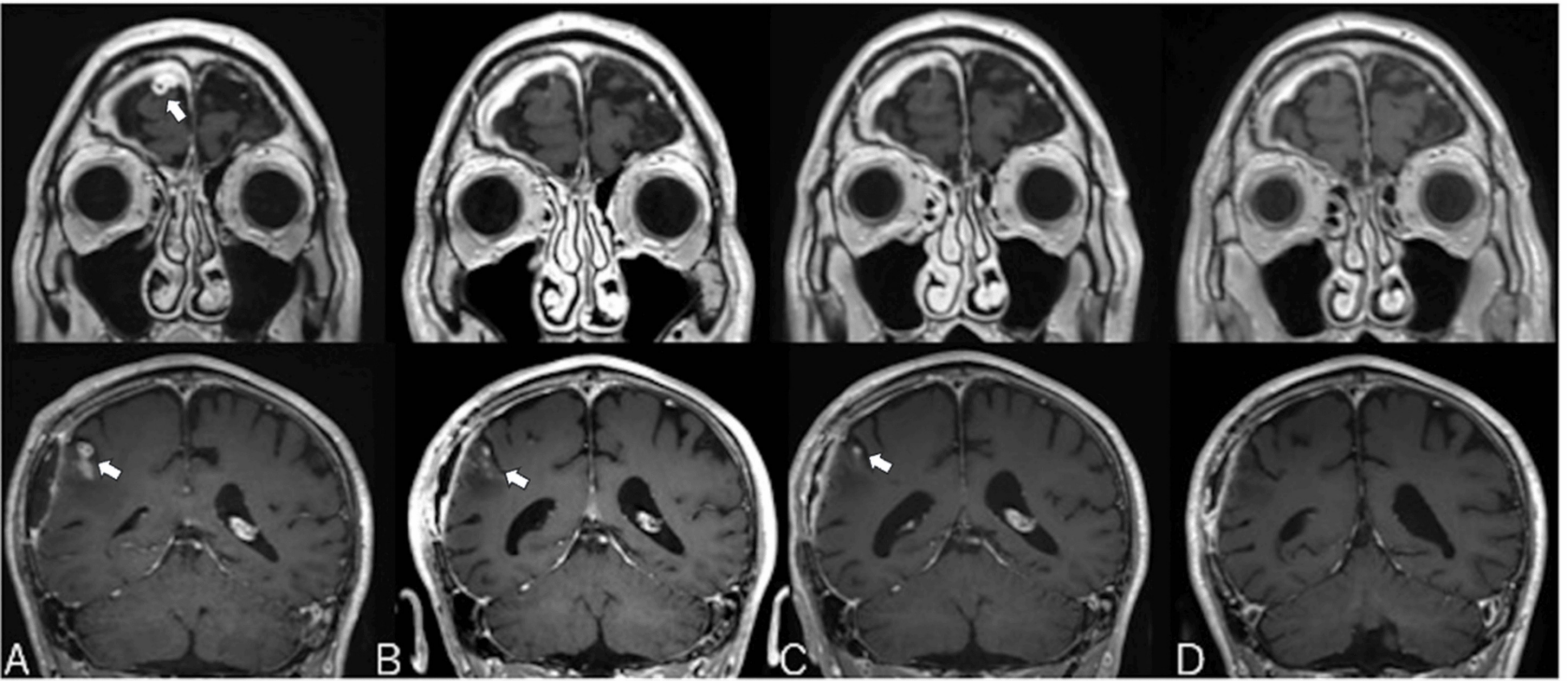
Coronal view of contrast-enhanced magnetic resonance image. (A) At six weeks after antifungal therapy initiation: shrinkage of the right temporal lobe lesion (arrow in the lower image), but a new lesion was detected in the right frontal lobe (arrow in the upper image). (B) At 12 weeks after antifungal therapy initiation: further reduction of the right temporal (arrow in the lower image) and frontal lobe lesions. (C) At two weeks after shifting to fluconazole (pre-discharge): further reduction of the right temporal (arrow in the lower image) and frontal lobe lesions. (D) At five weeks after shifting to fluconazole (post-discharge): absence of contrast-enhancing lesions in the right temporal and frontal lobes.

However, six weeks after treatment initiation, a new lesion was detected in the right frontal lobe (Figure 3A). Given the negative CSF cultures and the timing of the new lesion, the possibility of cryptococcal immune reconstitution inflammatory syndrome (IRIS) was considered. While IRIS is typically seen in immunosuppressed individuals, there are reports of its occurrence in immunocompetent patients. Although the patient did not have a known immunosuppressive condition, the clinical course suggests that IRIS could not be entirely ruled out. Consequently, the duration of L-AMB and 5-FC treatment was extended, further reducing the size of both the primary and new lesions.

After 12 weeks of treatment, and due to the favorable response of the lesions, the regimen was switched to fluconazole (FLCZ) (400 mg per day) (Figure 3B). Subsequently, at approximately 15 weeks after treatment initiation, the patient was discharged (Figure 3C). On follow-up cranial MRI, contrast-enhancing lesions in the right temporal and right frontal lobes were almost resolved (Figure 3D). The patient could return to work.

## Discussion

Central cryptococcal infection

Although cryptococcal infections are sometimes observed in patients with HIV, central cryptococcal infections in immunocompetent individuals are rare. [1,2] There have been 36 cases of immunocompetent patients with cryptococcoma (Table 1) [1-35]. Central cryptococcal infections in immunocompetent individuals often lead to a granulomatous reaction that results in a cryptococcoma. Regarding the cryptococcal variety, *Cryptococcus neoformans *typically causes meningitis in immunocompromised patients, whereas *Cryptococcus gattii *induces cryptococcoma in immunocompetent patients. Cryptococcomas have been observed in up to 69% of patients infected with *Cryptococcus gattii *[4,5].

**Table 1:** Summary of reported cases of intracranial cryptococcoma. This table summarizes previously reported cases of intracranial cryptococcoma, including patient demographics, lesion sites, initial symptoms, diagnostic findings, and treatment outcomes. The data highlights the variability in clinical presentation, diagnostic methods, and therapeutic approaches. CE: contrast enhancement, AMB: amphotericin B, 5-FC: flucytosine, FLCZ/FLC: fluconazole, CSF: cerebrospinal fluid, NA: not available/not applicable, CP angle: cerebellopontine angle, SMB: sulfamethoxazole, KOH: potassium hydroxide (used in fungal staining).

No.	Author/year	Sex/age	Lesion site	Initial symptoms	CE	biopsy/surgery	Culture	Antigen	India ink stain	Initial diagnosis	Treatment	Outcome
1	Chang CY et al., 2022 [3]	M/46	bilateral basal ganglia, Lung	headaches and difficulty walking		NA	+	＋(CSF)	+	Cryptococcal meningitis	AMB, 5-FC, FLC, Dexamethasone, VP shunt	resolved, no relapse
2	Popovich et al., 1990 [8]	F/47	Rt ventricle	headache, nausea	NA	NA	+	NA	NA	NA	surgery, AMB, 5-FC	resolved
3	Yu et al.,1995 [9]	M/31	pituitary gland	polydipsia, polyuria (no CNS sign)	Y	+	NA	+ (CSF)	NA	pituitary adenoma	biopsy, AMB, 5-FC	lesion smaller
4	Nucci et al., 1995 [10]	F/29	basal ganglia, cerebellum, Rt ventricle	sleepiness, vomiting	N	+ (autopsy)	NA	NA	NA	cysticercosis tenasia	dexamethasone	death
5	Caldemeyer et al., 1997 [11]	F/11	posterior fossa, multiple	back pain, lethargy	R	+	-	-	-	NA	surgery, AMB	NA
6	Kesler et al., 1999 [12]	M/16	Rt pons	cranial nerve and tract deficit	R	not performed	-	+ (CSF)	+	NA	antifungal	resolved
7	Krishnan et al., 2004 [13]	M/72	Lt parietal lobe Rt cerebellum	depression, confusion	Y	+ (from skin)	NA	+ (serum)	+	NA	AMB, FLC	death
8	Colom et al., 2005 [14]	M/60	basal ganglia	headache, somnolence	NA	+	+	+ (CSF and serum)	NA	NA	biopsy, AMB, 5-FC	asymptomatic
9	Ho et al., 2005 [15]	F/55	Rt frontal lobe	headache, facial palsy	R	+	-	+ (serum)	NA	tumor	surgery, AMB, FLC	not recurrent
10	Nadkarni et al., 2005 [16]	M/22	parietal lobe	seizure	R	NA	NA	NA	NA	tuberculoma	biopsy, surgery, AMB	seizure free
11	Saigal et al., 2005 [17]	M/49	bilateral basal ganglia	headache, syncope, mental change	R	not performed	-	+ (serum)	+	NA	AMB, 5-FC, FLCZ	lesion resolved
12	Oliveira et al., 2007 [18]	M/64	Rt temporal lobe	fever, anorexia, disorientation	N	+ (autopsy)	-	+ (CSF and serum)	NA	NA	aspiration, AMB	death
13	Gologrsky et al., 2007 [19]	M/11	Rt cerebellum	headache, emesis	R	+ (KOH)	-	+ (serum)	NA	vascular or tumor	surgery, AMB, 5-FC, FLC	normal
14	Kocaeli et al., 2008 [20]	F/54	Lt basal ganglia, Rt midbrain, Lt parietal lobe	headache, dysphagia, speech disorder, seizure	Y	+	+	NA	NA	metastasis	biopsy, antifungal	death
15	McMahon et al., 2008 [21]	F/68	Lt pons, middle cerebellum	falls, torsardes de pointes	NA	+	NA	NA	NA	NA	biopsy, AMB, 5-FC, FLC	death
16	Patro et al., 2009 [2]	F/52	Rt cerebellum (PC Angle)	giddiness, tinnitus, headache, vomiting	R	+	NA	NA	+	tumor, tuberculous	surgery, antifungal	recovered
17	Patro et al., 2009 [2]	M/23	cerebellum	double vision, headache, slurred speech	R	-	-	+ (CSF)	NA	tuberculous	SMB, 5-FC	resolved
18	Eghwrudjakpor et al., 2009 [22]	F/10	Lt parieto-occipital lobe, Lt frontal lobe	febrile, headache, seizure, impaired consciousness	R	+	NA	NA	+	NA	ketoconazole, surgery, antifungal medication	death
19	Li et al., 2010 [23]	F/49	Rt occipital lobe	headache, vomiting	R	+	NA	NA	-	Tumor	surgery, AMB	resolved
20	Tore et al., 2010 [24]	F/54	Lt basal ganglia, mid brain	headache, snoring, smelling unpleasant odors	R	+	-	NA	+	NA	AMB, 5-FC	Death
21	Chen et al., 2011 [25]	M/47	brain stem	NA	R	+	NA	NA	NA	tumor (glioblastoma)	NA	NA
22	Batista et al., 2012 [26]	F/37	Rt fronto-parietal lobe	headache, seizure	R	+	NA	NA	NA	Tumor	AMB	NA
23	Zhu et al., 2013 [27]	F/1	Rt parieto-occipital lobe	seizure	NA	+	-	NA	-	vascular malformation	surgery, FLC	resolved
24	Hagan et al., 2014 [28]	F/25	Lt basal ganglia	right paresthesia and muscle weakness	R	+	-	+ (CSF)	-	tumor (glioblastoma)	AMB, FLC	no recurrence
25	Hur et al., 2015 [29]	M/47	Lt pons	headache, nausea	R	+	NA	NA	NA	NA	surgery, antifungal medication (AMB)	resolved
26	Paiva et al., 2017 [30]	F/54	Lt occipital lobe	mental confusion	R	+	+	NA	NA	tumor	surgery, AMB, FLC	death
27	Ulett et al., 2017 [5]	M/55	Rt frontal lobe	headache	R	+	-	+ (CSF and serum)	NA	tumor (glio-blastoma)	Dexamethasone, AMB, 5-FC, surgery	resolved
28	Ang et al., 2017 [1]	M/59	Rt frontal lobe	left-side weakness	Y	+	-	+ (serum)	NA	Metastasis	AMB, 5-FC, FLCZ	resolved
29	Kelly et al., 2018 [6]	F/19	Lt frontal lobe, Lt temporal lobe	headache, vomiting	R	+	NA	NA	NA	NA	surgery, FLC	resolved
30	Akyeampong et al., 2019 [31]	M/30	Rt frontoparietal lobe, parieto-occipital lobe	seizure	R	+	-	-	NA	NA	surgery	resolved
31	Salvador et al., 2019 [32]	M/26	Lt parietal lesion	seizure, headache, diplopia	Y	+	NA	NA	NA	neoplasia	surgery	NA
32	Misra et al., 2020 [33]	M/55	Lt frontal lobe	difficulty in speaking, convulsion	R	+	NA	+ (CSF)	+	Metastasis	surgery, AMB, 5-FC, FLCZ	NA
33	Brunasso et al., 2021 [4]	M/32	Rt temporal lobe	seizure	R	+	-	NA	NA	tumor (glioma)	surgery	resolved
34	Boa Sorte et al., 2022 [7]	M/64	Rt temporal lobe	headache, visual change	R	+	NA	NA	NA	malignant glioma	surgery, AMB, 5-FC	resolved
35	Malhotra et al., 2023 [34]	M/52	Lt frontoparietal lesion	headache, vomiting, vison change, seizure	Y	not performed	-	+ (CSF)	NA	NA	AMB, 5-FC, FLCZ	NA
36	Li et al., 2023 [35]	M/40	Rt frontal lobe	headache, acute hemiplegia (infarction)	R	+	NA	NA	NA	high grade brain tumor	AMB, 5-FC, FLCZ	no recurrence

Symptoms of central cryptococcal infection

Central cryptococcal infections typically manifest with fever, headache, nausea, and impaired consciousness [11]. According to the literature (Table 1), headache is the most prevalent symptom, with 20 patients reporting it during their initial presentation. Among 36 cases, six patients presented with stroke-like symptoms, including paralysis, weakness, sensory disturbances, and cranial nerve abnormalities; of these, four patients also experienced headaches. Meanwhile, cases of cryptococcoma presenting with stroke-like symptoms without fever or headache are rare.

Diagnosis of central cryptococcal infection

Diagnosing central cryptococcal infection involves culture, antigen testing, staining, and histopathological analysis. Culture and antigen tests are highly sensitive and are the gold standard for diagnosing cryptococcal infections [23]. However, in immunocompetent patients who are at risk for cryptococcoma development, cryptococcal antigen, culture, and staining are less likely to be positive [5]. Li et al. suggested that this phenomenon may be attributed to the localization of yeasts within cryptococcomas, potentially lowering the test’s sensitivity when using serum and cerebrospinal fluid [23]. According to a literature review, of the 36 cases of cryptococcoma or brain abscess in immunocompetent patients, cultures, CSF or serum antigen, and staining were positive in only 5, 15, and 8 cases, respectively.

Therefore, in cases of cryptococcoma formation, antigen testing and culture are unlikely to return positive results. In contrast, histopathology confirmed the diagnosis in 30 of 36 cases. The reason why a biopsy is performed in several cases is the recognition that immunocompetent patients are less likely to have brain abscesses and that cryptococcal antigen and culture tests are less likely to have positive results. Additionally, ring-enhancing lesions on MRI are often misdiagnosed as tumors on imaging. In the literature, ring-enhancing lesions were present in 23 cases, and 16 were misdiagnosed as brain tumors; brain abscess or cryptococcoma were not suspected in any case. Thus, when a central cryptococcal infection develops in an immunocompetent patient, considering a brain abscess or cryptococcoma is unlikely because the antigen and culture tests are normally negative, and MRI often shows ring-enhancing lesions.

Jeong et al. described a case of a brain abscess that was initially misdiagnosed as cerebral infarction based on symptoms and imaging findings [36]. However, in addition to paralysis, fever (38.7°C), which may have been a clue to conduct additional examinations, was noted in the patient.

Clues on imaging examinations to avoid misdiagnosis include a larger FLAIR high-signal area compared with the DWI high-signal lesion, signal changes on follow-up MRI, and DWI high-signal that does not coincide with the area dominated by the vessel. It may be difficult to diagnose a central cryptococcal infection from symptoms or antigen and culture tests when the patient does not have typical symptoms or when cryptococcoma forms in an immunocompetent patient. There is a risk of misdiagnosis as brain infarction on DWI or brain tumor on contrast-enhanced MRI.

Our patient presented with two episodes of transient right upper limb paralysis: during the initial visit, MRI revealed no cerebral infarction, leading to a diagnosis of TIA; during the subsequent visit, a small cerebral infarction was detected in the left hemisphere (evidenced by high DWI signal, low ADC, and no contrast enhancement), which was managed with antiplatelet agents. Cervical echocardiography performed during hospitalization showed mild plaques. Upon the third visit, the patient exhibited left upper limb paralysis and a ring-enhancing lesion in the right cerebral hemisphere. Based on these findings, the first two episodes were deemed cerebrovascular, while the third symptom was attributed to central cryptococcal infection.

Although cerebral infarction caused by *Cryptococcoma* has been reported, it has been postulated to result from large vessel invasion by *Cryptococcoma*. Given the absence of evidence for large vessel invasion, the absence of recurrences of left hemisphere image abnormalities, and the absence of recurrences of right-sided neurological symptoms in this case, it is thought that the previous two episodes were not caused by cryptococcoma but were simply episodes of stroke. The third episode, however, is considered to be due to brain compression from cryptococcoma, based on the subsequent clinical course and imaging findings.

Treatment and prognosis of central cryptococcal infection

Guidelines for the antifungal treatment of central cryptococcal infection recommend intravenous amphotericin B (L-AMB) and fluconazole (5-FC) for at least six weeks, followed by oral fluconazole for 6-18 months, ideally initiated immediately following diagnosis [37,38]. In immunocompetent patients, prior studies have indicated a mortality rate of 15-25% for central cryptococcal infections [39]. The prognosis appears to be poorer in cases with delayed diagnosis or lesions extending into the brainstem. Among the 36 cases of central cryptococcal infection we investigated, seven resulted in mortality, yielding a mortality rate of 20%. Delayed diagnosis and treatment are associated with poor prognosis, and in the worst cases, patient deaths have been reported. It shows that even cryptococcoma in immunocompetent patients can lead to death if treatment is delayed. In our case, blood tests (β-D glucan), blood culture, and cerebrospinal fluid culture returned negative results, and the diagnosis was only confirmed through histopathological analysis. If a rapid diagnosis fails to identify a neoplastic lesion, and a central cryptococcal infection is suspected, it is crucial to shift the strategy from excision to biopsy only and initiate early antifungal therapy. While reports suggest the effectiveness of lesion excision, excision could worsen postoperative symptoms in cases such as ours where the lesion was located in the postcentral gyrus. Therefore, confirming the presence of a cryptococcoma and subsequently administering appropriate antibiotic therapy is likely to result in the best outcome.

In our case, the early initiation of antifungal therapy allowed the patient to resume his daily activities.

## Conclusions

Without being misled by the medical history (repeated TIA/stroke, no history of immunodeficiency disease), detailed history-taking and accurate evaluation of the MRI demonstrate the possibility of early therapeutic intervention for cryptococcoma, which could have resulted in death if treatment had been delayed, even in immunocompetent patients. This case highlights the critical role of tissue biopsy in establishing a diagnosis, especially in immunocompetent individuals, where obtaining a positive fungal culture can be challenging. Since cerebrospinal fluid cultures are often negative in such cases, histopathological examination remains a key diagnostic tool to confirm cryptococcal infection and guide appropriate antifungal treatment.
